# Micropillar-induced changes in cell nucleus morphology enhance bone regeneration by modulating the secretome

**DOI:** 10.21203/rs.3.rs-5530535/v1

**Published:** 2025-01-07

**Authors:** Xinlong Wang, Yiming Li, Zitong Lin, Indira Pla, Raju Gajjela, Basil Baby Mattamana, Maya Joshi, Yugang Liu, Huifeng Wang, Amy B. Zun, Hao Wang, Ching-Man Wai, Vasundhara Agrawal, Cody L. Dunton, Chongwen Duan, Bin Jiang, Vadim Backman, Tong-Chuan He, Russell R. Reid, Yuan Luo, Guillermo A. Ameer

**Affiliations:** 1 Center for Advanced Regenerative Engineering, Northwestern University, Evanston, IL 60208, USA; 2 Department of Biomedical Engineering, Northwestern University, Evanston, IL 60208, USA; 3 Department of Preventive Medicine, Northwestern University Feinberg School of Medicine, Chicago, IL 60611, USA; 4 Proteomics Center of Excellence, Northwestern University, Evanston, IL 60208, USA; 5 Molecular Oncology Laboratory, Department of Orthopedic Surgery and Rehabilitation Medicine, The University of Chicago Medical Center, Chicago, IL 60637, USA; 6 Center for Genetic Medicine, Northwestern University Feinberg School of Medicine, Chicago, IL 60611, USA; 7 Center for Physical Genomics and Engineering, Northwestern University, Evanston, IL 60208, USA; 8 Department of Surgery, Northwestern University Feinberg School of Medicine, Chicago, IL 60611, USA; 9 Chemistry of Life Process Institute, Northwestern University, Evanston, IL 60208, USA; 10 Laboratory of Craniofacial Biology and Development, Section of Plastic and Reconstructive Surgery, Department of Surgery, The University of Chicago Medical Center, Chicago, IL 60637, USA; 11 Northwestern University Clinical and Translational Sciences Institute, Northwestern University Feinberg School of Medicine, Chicago, IL 60611, USA; 12 Center for Collaborative AI in Healthcare, Institute for AI in Medicine, Northwestern University Feinberg School of Medicine, Chicago, IL 60611, USA; 13 International Institute for Nanotechnology, Northwestern University, Evanston, IL 60208, USA; 14 Simpson Querrey Institute for Bionanotechnology, Northwestern University, Chicago, IL 60611, USA

## Abstract

Nuclear morphology, which modulates chromatin architecture, plays a critical role in regulating gene expression and cell functions. While most research has focused on the direct effects of nuclear morphology on cell fate, its impact on the cell secretome and surrounding cells remains largely unexplored, yet is especially crucial for cell-based therapies. In this study, we fabricated implants with a micropillar topography using methacrylated poly(octamethylene citrate)/hydroxyapatite (mPOC/HA) composites to investigate how micropillar-induced nuclear deformation influences cell paracrine signaling for osteogenesis and cranial bone regeneration. *In vitro*, cells with deformed nuclei showed enhanced secretion of proteins that support extracellular matrix (ECM) organization, which promoted osteogenic differentiation in neighboring human mesenchymal stromal cells (hMSCs). In a mouse model with critical-size cranial defects, nuclear-deformed hMSCs on micropillar mPOC/HA implants elevated Col1a2 expression, contributing to bone matrix formation, and drove cell differentiation toward osteogenic progenitor cells. These findings indicate that micropillars not only enhance the osteogenic differentiation of human mesenchymal stromal cells (hMSCs) but also modulate the secretome, thereby influencing the fate of surrounding cells through paracrine effects.

## Introduction

The nucleus is a dynamic organelle that changes its morphology in response to the cell’s status.^1^ Its morphology has critical influence on nuclear mechanics, chromatin organization, gene expression, cell functionality and disease development.^2–5^ Abnormal nuclear morphologies, such as invagination and blebbing, have functional implications in several human disorders, including cancer, accelerated aging, thyroid disorders, and different types of neuro-muscular diseases.^6,7^ In addition, severe nuclear deformation is also observed during tissue development, cell migration, proliferation, and differentiation.^2^ Several structural components within the nucleus—including the nuclear envelope, lamins, nuclear actin, and chromatin—work together to determine its shape and structure.^8^ Although the underlying mechanisms are not yet fully understood, nuclear deformation has been found to affect cell behaviors through mechanotransduction processes.^9^ In addition, nuclear morphological changes have been reported to affect nuclear membrane tension and unfolding, which regulate the structure of the nuclear pore complex.^10^ This, in turn, influences the nuclear shuttling of transcription factors (e.g., YAP) and ions (e.g., Ca^2+^), ultimately impacting cell functions.^11,12^ In our previous study, we demonstrated that altering nuclear morphology using micropillar topography affects nuclear lamin A/C assembly, which, in turn, influences chromatin tethering, packing, and condensation.^13^ These changes affect transcriptional accessibility and responsiveness, thereby regulating gene expression and stem cell differentiation.

To manipulate nuclear morphology, various biophysical tools have been developed, including atomic force microscopy (AFM) nanoindentation, optical, magnetic, and acoustic tweezers, microfluidic devices, micropipette aspiration, plate compression, substrate deformation, and surface topography modulation.^14–21^ Among these methods, regulating the surface topography of materials is more accessible and has broader implications for regenerative engineering. One commonly used approach is the fabrication of pillar structures, which are employed to deform cell nuclei and study nuclear properties such as mechanics and deformability.^22^ These micropillar designs have been utilized to manipulate various cell functions, including migration, adhesion, proliferation, and differentiation.^23–26^ A wide range of materials can be used to create these structures, such as poly-L-lactic acid (PLLA), poly(lactide-co-glycolide) (PLGA), OrmoComp (an organic-inorganic hybrid polymer), and methacrylated poly(octamethylene citrate) (mPOC).^13,26–28^ Among these options, mPOC is particularly suitable for bone regeneration due to its major component, citrate, which acts as a metabolic factor to enhance the osteogenesis of mesenchymal stromal cells (MSCs).^29^

Although the influence of nuclear morphogenesis on the functions of individual cells is being intensively investigated, its role in regulating cellular secretion remains unclear. Bioactive molecules secreted by cells are crucial for intercellular communication, affecting various biological processes such as inflammation, cell survival, differentiation, and tissue regeneration.^30,31^ The success of many cell and exosome-based therapies relies on the cellular secretome. In this study, we fabricated micropillars to manipulate nuclear morphology and investigated their effects on the secretome of human mesenchymal stromal cells (hMSCs). We incorporated hydroxyapatite (HA), the primary inorganic component of native bone tissue, with micropatterned methacrylated poly(octamethylene citrate) (mPOC) to create the micropillars, promoting bone formation. Our results showed that mPOC/HA micropillars facilitated osteogenic differentiation of hMSCs compared to flat mPOC/HA samples *in vitro*. Secretome analysis revealed that hMSCs with deformed nuclei exhibited higher expression levels of bioactive factors associated with extracellular matrix (ECM) components and organization, as well as ossification. *In vivo*, both mPOC/HA flat and micropillar scaffolds seeded with hMSCs resulted in new bone formation; however, the micropillar group demonstrated significantly greater new bone volume and regenerated tissue thickness. Spatial transcriptomic analysis further confirmed elevated expression of genes related to the regulation of ECM structures, consistent with the secretome analysis results. These findings suggest that the influence of nuclear deformation on the osteogenesis of hMSCs operates through similar mechanisms in both *in vitro* and *in vivo* environments. Therefore, microtopography engineering of scaffold to control nuclear morphology is a promising approach to enhance bone regeneration.

## Results

### Influence of micropillar structures on physical and chemical properties of mPOC/HA implants

**Figure 1.**
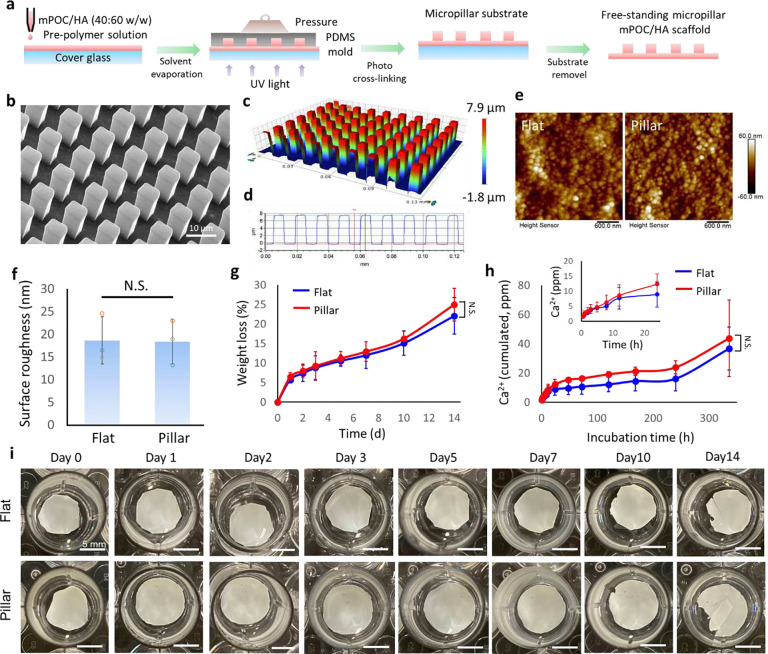
Fabrication of surface engineered mPOC/HA implants. **a.** Illustration shows the combination of UV lithography and contact printing to fabricate free-standing mPOC/HA micropillars. **b.** SEM image shows the micropillar structures made of mPOC/HA. **c.** Optical microscope image and **d.** cross-section analysis of mPOC/HA micropillars. **e.** Surface scanning of flat and micropillar implants by AFM. **f.** Surface roughness of flat and micropillar implants. N.S., no significant difference, n = 3 biological replicates. **g.** Degradation test and **h.** calcium release of flat and micropillar mPOC/HA implants. N.S., no significant difference, n = 4 biological replicates, insert plot shows the initial release of calcium within 24 h. **i.** Representative images of flat and micropillar implants at different time points after accelerated degradation.

mPOC prepolymer was synthesized according to our previous report,^32^ and its successful synthesis was confirmed via the nuclear magnetic resonance (1H NMR) spectrum (**Fig. S1a-c**). The size of HA nanoparticles is around 100 nm, as characterized by dynamic light scattering (DLS) (**Fig. S1d**). To mimic the nature of bone composition,^33^ 60% (w/w) HA was mixed with mPOC, and the slurry was used to fabricate flat and micropillar implants using a combination of UV lithography and the contact printing method (Fig. 1a). The square micropillars, with dimensions of 5 by 5 in side length and spacing, were fabricated (Fig. 1b). The height of the micropillars is around 8 μm, which can cause significant nuclear deformation (Fig. 1c,d).^27^ Fourier transform infrared (FTIR) spectrum shows a similar typical peak of functional groups in mPOC and mPOC/HA implants (**Fig. S1e**). The surface roughness of the implants was scanned using an atomic force microscope (AFM) (Fig. 1e). The analysis result indicates that the topography didn’t affect the surface roughness of the implants (Fig. 1f). Additionally, we tested the hydrophilicity of flat and micropillar implants via water contact angle measurement (**Fig. S2**). Although, at the initial state, the flat surface was more hydrophilic, there was no significant difference in the water contact angle after a 5-minute stabilization process.

The mechanical properties of the implants were tested using the nano-indentation method. The force-indentation curve of the flat sample has a sharper slope, indicating it is stiffer than the micropillar sample (**Fig. S3a**). The Young’s Modulus of the flat sample (0.95 ± 0.12 GPa) is significantly higher than that of the micropillars (0.48 ± 0.02 GPa) and the lateral modulus of the micropillars (46.88 ± 1.49 MPa) (**Fig. S3b,c**). However, based on a previous report, the high modulus of the substrates is beyond the threshold that cells can distinguish and does not have an influence on nuclear morphology manipulation.^34,35^ Accelerated degradation and calcium release tests of the implants were performed in DPBS at 75 °C with agitation.^36^ There is a burst weight loss and calcium release of both flat and micropillar samples at day 1, followed by a gradual change until day 10, and another increase in the degradation and calcium release rate from day 10 to 14 (Fig. 1g,h). The micropillar structure enhanced the degradation and calcium release, but not significantly. According to the images of the samples captured at different time points, the initial burst degradation and calcium release can be attributed to the fast surface erosion of both scaffolds, as many small pores can be observed on their surfaces. From day 10 to 14, scaffolds started break into pieces that may lead to another burst degradation and calcium release (Fig. 1i).

### Nuclear deformation facilitates osteogenic differentiation of hMSCs

**Figure 2.**
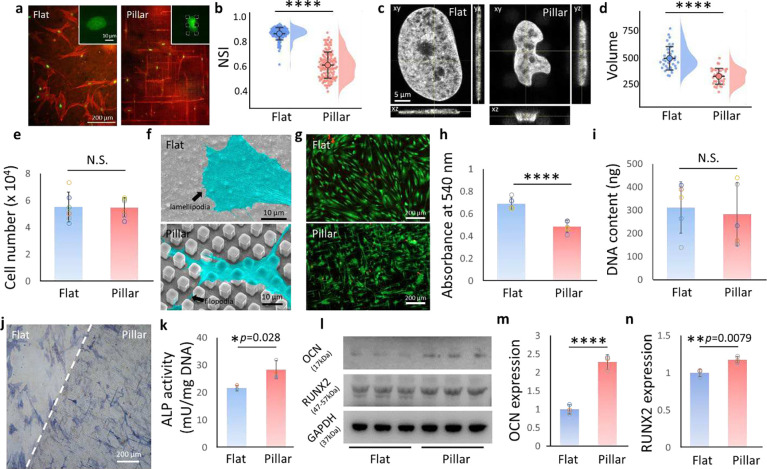
Nuclear deformation promotes osteogenic differentiation of hMSCs. **a.** Staining of nucleus (green) and F-actin (red) of hMSCs on flat and micropillar mPOC/HA surfaces. Insert: high magnification of cell nucleus. Dashed lines indicate micropillars. **b.** Analysis of nuclear shape index of hMSCs. n= 117 (flat) and 132 (pillar) collected from 3 biological replicates, ****p<0.0001. **c.** Orthogonal view of cell nucleus on flat and micropillar surfaces. **d.** Nuclear volume analysis based on 3D construction of the confocal images of cell nuclei. n= 35 cells collected from 3 biological replicates, ****p<0.0001. **e.** Initial cell attachment on flat and micropillar surfaces. n=5 biological replicates, N.S., no significant difference. **f.** SEM images show the cell attachment on flat and micropillar mPOC/HA surfaces. **g.** Live/dead staining of hMSCs on flat and micropillar surfaces at 72 h in osteogenic medium. **h.** Cell metabolic activity of cells on flat and micropillar surfaces tested by a MTT assay. n=5 biological replicates, ****p<0.0001. **i.** Cell proliferation tested via DNA content after 72 h induction. n=5 biological replicates, N.S., no significant difference. **j.** ALP staining of hMSCs on flat and micropillar surfaces after 7 d induction. **k.** ALP activity test of cells after 7 d osteogenic induction. n=3 biological replicates. **l.** Blot images of osteogenic marker OCN and RUNX2 in cells cultured on flat and micropillar implants. GAPDH is shown as a control. Quantification **m.** OCN and **n.** RUNX2 according to western blot tests. n=3 biological replicates, ****p<0.0001.

hMSCs were cultured on the flat and micropillar mPOC/HA surfaces in osteogenic medium and stained for F-actin and nuclei after 3 days (Fig. 2a). Noticeable deformation in both the nucleus and cytoskeleton was observed, consistent with mPOC micropillars.^13^ The Nuclear shape index (NSI) was calculated to assess the degree of nuclear deformation.^27^ A significantly lower NSI value, indicating more severe deformation, was found in the micropillar group (Fig. 2b). Confocal images were then employed to evaluate the 3D geometry of cell nuclei (Fig. 2c). 3D reconstruction analysis revealed that several geometric parameters, including nuclear volume, surface area, and project area, were significantly decreased on micropillars, while nuclear height was significantly increased (Fig. 2d and **Fig. S4**).

We then investigated the impact of micropillars on cell adhesion, a crucial aspect for manipulating cell function.^37^ Initial cell attachment tests revealed that the micropillar structure did not influence cell attachment on the implants (Fig. 2e). SEM imaging of cell adhesion demonstrated that cells formed lamellipodia on flat surfaces but exhibited more filopodia on micropillars (Fig. 2f). Filopodia were observed on the top, side, and bottom of micropillars, indicating that cells were sensing the 2.5D environment using these antennae-like structures.^23^ The majority of cells were found to be viable on both flat and micropillar substrates, as evidenced by live/dead staining (Fig. 2g and **Fig. S5**). While the micropillars reduced cell metabolic activity (Fig. 2h), there was no significant impact on cell proliferation after 3 days of culture (Fig. 2i).

To assess the impact of mPOC/HA micropillars on the osteogenesis of hMSCs, we stained ALP (alkaline phosphate) on a substrate with a combination of half flat and half micropillar structures (Fig. 2j). Quantification results demonstrated a significant increase in ALP activity on the micropillars (Fig. 2k). Furthermore, additional osteogenic differentiation markers of hMSCs, including RUNX2 and osteocalcin (OCN), were quantified through western blot analysis (Fig. 2l). The quantification of these proteins revealed a significant increase in both RUNX2 and OCN in cells on micropillars, confirming that the structures can effectively promote the osteogenic differentiation of hMSCs (Fig. 2m,n).^13,26,27^

### Micropillars modulate the secretome of hMSCs that regulate extracellular matrix formation.

**Figure 3.**
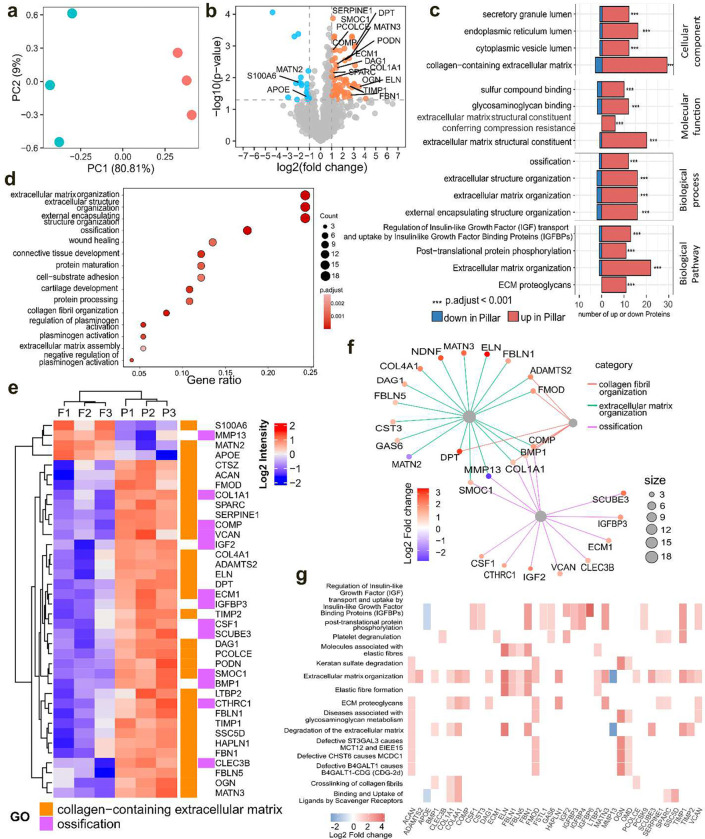
Secretome of hMSCs on flat and micropillar mPOC/HA surfaces. **a.** PCA plot of differentially expressed proteins secreted by hMSCs on flat and micropillars. Cyan: flat; Red: micropillar. **b.** Volcano plot of proteins secreted by hMSCs seeded on micropillars compared to the flat surface. Blue dots and orange dots indicate significantly downregulated and upregulated proteins secreted by cells on micropillars compared to those on flat surface. Grey dots indicate non-significantly changed proteins. A threshold of expression greater than 2 times fold-change with *p*<0.05 was considered to be significant. Proteins that are related with collagen-ECM pathways are labelled. **c**. Top 4 significantly enriched GO and Pathways based on their adjusted p-values. **d.** The most significant enriched GO terms of the biological domain with respect to biological process. **e.** Heatmap of proteins that are related with collagen-containing extracellular matrix and ossification. F indicates flat samples and P indicates pillar samples, n=3 biological replicates for each group. **f.** The linkages of proteins and GO terms in biological process related with collagen fibers, ECM, and ossification as a network. **g**. Heatmap of top 15 enriched terms plotted based on Reactome pathway analysis.

Previously, we demonstrated the ability of micropillar implants to enhance *in vivo* bone formation.^13^ However, the newly formed bone was not in close contact with the implant. Consequently, we hypothesized that nuclear deformation on micropillars might impact cellular secretion, thereby influencing osteogenesis through paracrine effects. To test this hypothesis, secretome analysis was conducted using medium collected from flat and micropillar samples. Differences in protein secretion levels between the two groups were depicted through principal component analysis (PCA) and a volcano plot, revealing a significant influence of nuclear deformation on the secretome (Fig. 3a,b). Gene ontology (GO) analysis was performed to annotate the significantly altered proteins in relevant processes.^38^ Top changes in cellular component, molecular functions, biological processes, and biological pathways indicated that micropillars predominantly affected extracellular matrix (ECM)-related processes (Fig. 3c and **Fig. S6–8**). Moreover, ossification and collagen fibril organization were identified as biological processes significantly overrepresented by differentially expressed proteins (Fig. 3d). The heatmap plot of proteins associated with collagen-containing extracellular matrix and ossification showed predominant upregulation on micropillars (Fig. 3e). The linkages of proteins and GO terms in biological process highlighted that ECM organization forms the largest cluster and is closely associated with the ossification process (Fig. 3f).

Reactome pathway analysis was further conducted to assess potential downstream effects of secretome changes on micropillars.^39^ Results indicated that pathways related to ECM organization, ECM proteoglycans, and collagen fibril crosslinking were among the top 15 pathways significantly overrepresented by differential expressed pathways (DEP), predominantly showing upregulation (Fig. 3g and **Fig. S9**). We also noticed an upregulation in the degradation of the ECM on micropillars, indicating enhanced ECM remodeling which a crucial factor for tissue regeneration.^40^ These findings suggest that micropillars can influence the ECM formation of hMSCs through paracrine effects. Additionally, we performed proteomic analysis using cells cultured on flat and micropillar mPOC/HA scaffolds (**Fig. S10**). PCA and volcano plots indicated significant influences of nuclear deformation on protein expression. Pathway analysis revealed significant changes in many cell proliferation-related processes, consistent with previous transcriptomic tests on micropillars.^13^

### Nuclear deformed cells facilitate osteogenic differentiation of undeformed cells by affecting ECM.

**Figure 4.**
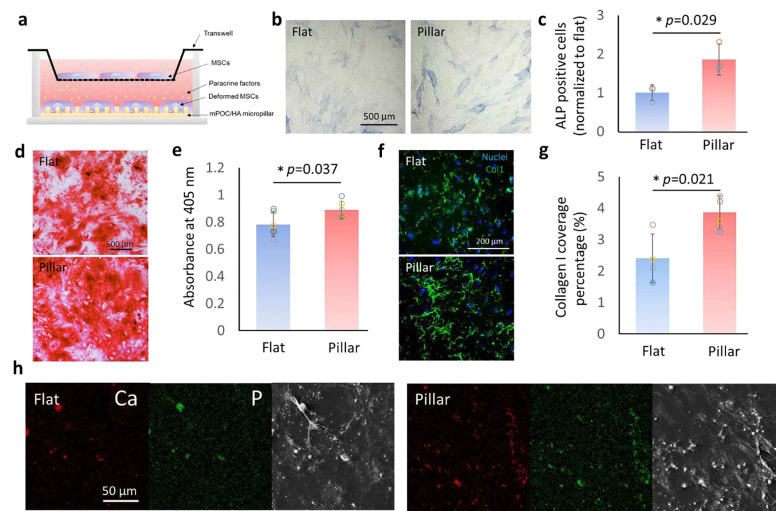
The paracrine effect of cells with/without nuclear deformation tested through transwell assay. **a.** Schematic illustration of the experiment setup. **b.** ALP staining and **c.** quantification of ALP positive cells on transwell membrane incubated with undeformed and deformed MSCs (n=3). **d.** ARS staining and **e.** quantification of cells on transwell membrane incubated with undeformed and deformed MSCs (n=6). f. Immunofluorescence staining images of collagen in ECM of cells on transwell membrane incubated with undeformed and deformed MSCs. **g.** The coverage of collagen analyzed according to the staining images (n=4). **h.** EDS images showing Ca, P, and SEM images of cells on transwell membrane incubated with undeformed and deformed MSCs.

Since the micropillar surfaces can modulate the secretome of hMSCs, we investigated whether the deformed cells could influence the osteogenic differentiation of undeformed cells using a transwell assay (Fig. 4a). The flat and micropillar mPOC/HA surfaces were fabricated at the bottom of cell culture plates to manipulate the nuclear morphology of hMSCs, while undeformed hMSCs were seeded on a transwell membrane with 400 nm nanopores, allowing the exchange of growth factors. After cell attachment, all samples were cultured in osteogenic induction medium. ALP staining of the cells on the transwell membrane showed a higher number of ALP-positive cells when co-cultured with nuclear-deformed cells, indicating enhanced osteogenic differentiation (Fig. 4b,c). Additionally, Alizarin Red S (ARS) staining confirmed increased calcium deposition—a key step in osteogenesis—when the cells were cultured above the micropillar-treated cells (Fig. 4d,e). Based on the secretome analysis, hMSCs on micropillars appear to promote osteogenesis in the transwell culture by secreting proteins that enhance ECM structure and organization. Collagen staining revealed higher coverage, stronger staining intensity, and more interconnected collagen network structures in the transwell co-cultured with micropillar-treated cells (Fig. 4f,g). In addition, energy dispersive X-ray spectroscopy (EDS) images showed more Ca and P deposition in the transwell co-cultured with micropillar-treated cells (Fig. 4h). Together with the secretome analysis, these findings suggest that the proteins secreted by cells with deformed nuclei improve ECM organization in undeformed cells, thereby promoting osteogenesis.

#### mPOC/HA micropillar implant promotes bone formation *in vivo*

**Figure 5.**
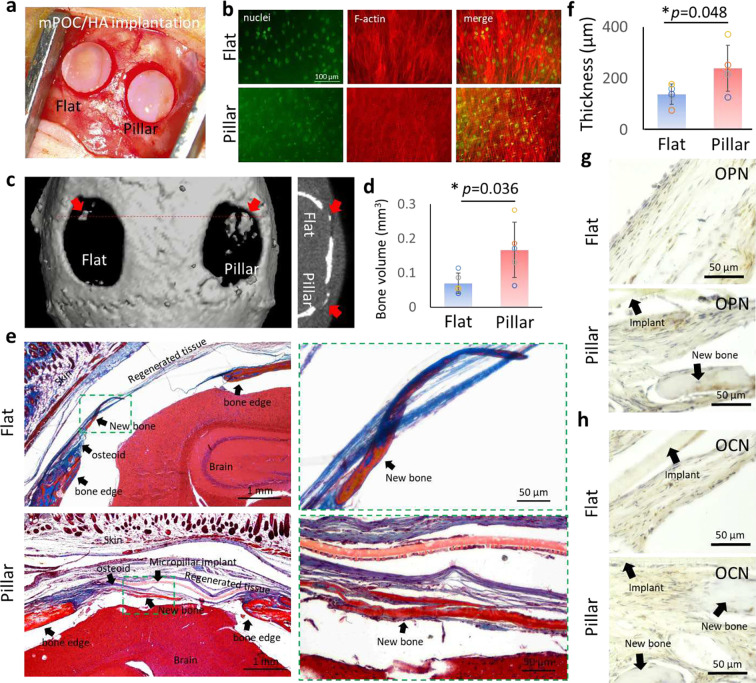
mPOC/HA micropillar implant promotes bone regeneration *in vivo*. **a.** Image shows implantation of hMSC seeded flat and micropillar mPOC/HA scaffolds. **b.** Staining images of nuclei (green) and F-actin (red) of cells on the implants. **c.** Representative μCT images of a typical animal implanted with hMSC-seeded flat (left) and micropillar (right) scaffolds at 12-weeks post-surgery. **d.** Regenerated bone volume in the defect region (n = 5 animals). **e.** Trichrome staining of the defect tissue treated with flat and micropillar implants. **f.** Average thickness of regenerated tissues with implantation of flat and micropillar scaffolds (n = 5 animals). IHC staining of osteogenic marker, **g.** OPN and **h.** OCN, in regenerated tissues with flat and micropillar implants.

To test the *in vivo* regeneration efficacy of mPOC/HA scaffolds, we created a critical size cranial defect model in nude mice. Two 4 mm diameter critical defects were made on the left and right sides of the skull tissue for the implantation of flat and micropillar scaffolds, respectively (Fig. 5a). The scaffolds were seeded with hMSCs for 24 hours to allow for cell attachment and nuclear deformation (Fig. 5b). After 12 weeks, micro CT was performed to evaluate the bone formation in the living animals. Based on the images, newly formed bone can be observed in the defect area with both flat and micropillar mPOC/HA implants (Fig. 5c and **Fig. S11**). Comparing this to our previous study using mPOC alone,^13^ the integration of HA clearly enhanced bone regeneration efficacy *in vivo*. Furthermore, larger bone segments were observed with the micropillar implant treatment. Quantification results confirmed a significantly increased bone volume with micropillar implant treatment (Fig. 5d).

Histology analysis was further performed to evaluate the influences of flat and micropillar mPOC/HA implants on bone regeneration. Trichrome staining images revealed that defects treated with micropillar implants exhibited more osteoid tissue (Fig. 5e and **Fig. S12**). Moreover, both flat and micropillar mPOC/HA implants showed evidence of newly formed bone tissue, indicating enhanced bone regeneration compared to the mPOC alone scaffold. As no bone segment was observed with flat mPOC implant treatment.^13^ The thickness of the regenerated tissue was quantified, and the results demonstrated a significant enhancement with micropillar implant treatment (Fig. 5f). Positive staining of osteogenesis markers, including osteopontin (OPN) and osteocalcin (OCN), was observed throughout the regenerated tissues with both flat and micropillar implants, indicating osteoid tissue formation (Fig. 5g,h). The tissue appeared more compact in the micropillar group compared to the flat group. Furthermore, regenerated bone segments were more frequently observed with micropillar implant treatment.

### Micropillar implants facilitated bone regeneration *in vivo* via regulation of ECM organization and stem cell differentiation.

**Figure 6.**
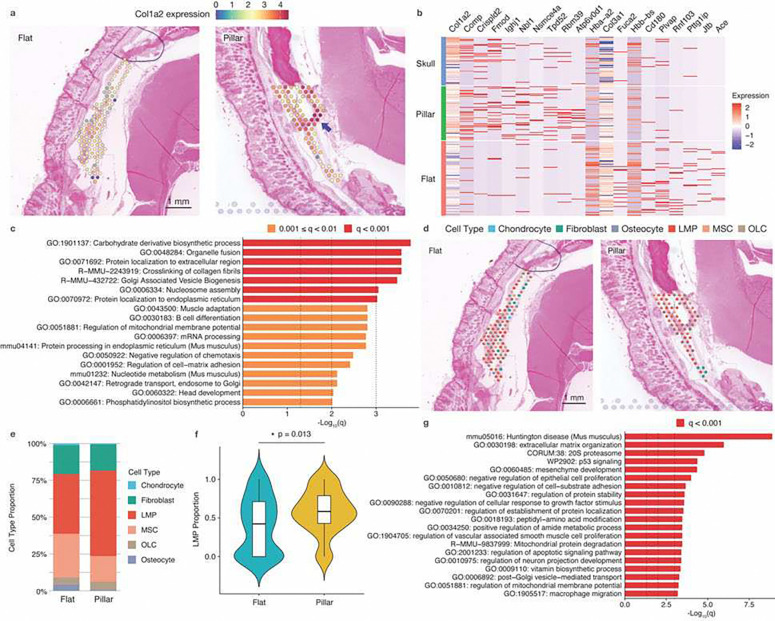
Spatial transcriptomic analysis of tissues regenerated with flat and micropillar implants. **a.** Spatial plot of Col1a2 expression profile in tissues regenerated with flat mPOC/HA implant and micropillar mPOC/HA implant. Arrow indicates enhanced expression around dura layer. **b.** The heatmap showing the top ten up- and down-regulated DEGs (pillar vs flat) in tissues regenerated with flat mPOC/HA implant, micropillar mPOC/HA implant, and native skull tissue. **c.** Gene Ontology analysis results based on the top 100 up-regulated genes (pillar vs flat). **d.** Deconvoluted cell types in each spatial capture location in flat and micropillar groups. Each pie chart shows the deconvoluted cell type proportions of the capture location. **e.** Bar plots of the cell type proportions in tissues regenerated with flat mPOC/HA implant and micropillar mPOC/HA implant. LMPs, MSCs, and fibroblasts are the predominant cell types. **f.** Violin plot of the proportion of LMPs in flat and micropillar groups. **g.** Top enriched processes associated with LMP compared with other cell lineages. LMP: late mesenchymal progenitor cells; MSC: mesenchymal stromal cells; OLC: MSC-descendant osteolineage cells

Histological analyses showed more new bone formation with micropillar implants, although the new bone tissue did not directly interact with the micropillar surfaces. To further investigate the transcription profile of the regenerated tissue, we performed spatial transcriptomics (ST) analyses with both flat and pillar samples (**Fig. S13**). ST represents a powerful tool to investigate the cellular environment and tissue organization by providing a detailed map of gene expression within the native tissue context.^41^ Differential gene expression (DGE) analysis revealed changes in expression levels between the two groups. Although only a few genes showed significant differences, all of them were related to ECM structure or organization (**Fig. S13**). Notably, the expression of Col1a2, critical for type I collagen formation (comprising 90% of the bone matrix), was enhanced in the micropillar group (Fig. 6a). This expression showed a gradient, increasing toward the dura layer, possibly due to the osteogenic contribution of dura cells.^42^ We then plotted a heatmap showing the top 10 up-regulated and down-regulated differentially expressed genes (pillar vs. flat) in comparison with those in native skull bone (Fig. 6b). The heatmap indicated that the tissue regenerated with micropillar implants had expression patterns more similar to native skull bone than the flat group. Gene Ontology (GO) analysis of DGEs was further performed to annotate their relevant biological processes (Fig. 6c). Protein localization to extracellular matrix and crosslinking of collagen fibrils were among the top 5 up-regulated processes in the micropillar group. These results are consistent with the secretome test, all indicating that micropillar structures can influence ECM organization via paracrine effects.

To further investigate the relationship between cell type composition and the regenerated tissues, we performed cellular deconvolution on the ST data using single-cell RNA sequencing (scRNA-seq) references from previously published studies.^43–45^ Several major cell lineages involved in bone regeneration were considered when deconvoluting the data (Fig. 6d). The most abundant cell type in regenerated tissues was late mesenchymal progenitor cells (LMPs), followed by MSCs and fibroblasts (Fig. 6e). There were also small proportions of MSC-descendant osteolineage cells (OLCs), osteocytes, osteoblasts, and chondrocytes. LMPs are identified as the late stage of MSCs through osteogenic differentiation.^43,46^ Among all cell types, the proportion of LMPs, which have high expression of marker genes associated with osteoblasts, was significantly increased in regenerated tissues with micropillar implants, indicating that these deformed cells facilitate the differentiation of MSCs toward the osteolineage (Fig. 6f). Additionally, GO analysis of DGEs (LMP versus other cell types) was performed to investigate the roles of LMPs in regenerated tissue. The results suggest that LMPs do not directly contribute to osteogenesis, a role performed by osteoblasts and osteocytes. Instead, LMPs can affect ECM formation, as the process of extracellular matrix organization is one of the top involved pathways (Fig. 6g). Thus, the results indicate that micropillar implants can facilitate skull tissue regeneration by promoting the differentiation of MSCs and ECM organization via paracrine effects.

## Discussion

Micropillars, as a typical topographical feature, have been extensively studied for their ability to regulate cell functions. Recent researches have shown that rigid micropillars can deform nuclear morphology, which in turn promotes the osteogenic differentiation of mesenchymal stem cells (MSCs), generating significant interest for bone regeneration applications.^26,27^ Our previous work demonstrated that mPOC micropillars enhanced bone regeneration in a mouse cranial defect model.^13^ The mPOC, a citrate-based biomaterial (CBB), is an excellent candidate for bone regeneration because citrate, an important organic component of bone, plays key roles in skeletal development and bone healing by influencing bone matrix formation and the metabolism of bone-related cells.^47^ In this study, hydroxyapatite (HA) was incorporated into mPOC to further enhance its regenerative potential, leveraging HA’s well-known osteoconductive properties.^48^ Both *in vitro* and *in vivo* experiments confirmed that the addition of HA significantly improved bone regeneration compared to mPOC alone.^13^ Moreover, several products made from CBB/HA composites have recently received FDA clearance, highlighting the promising clinical potential of mPOC/HA micropillars for bone regeneration applications.^49^

Despite recent intensive investigations into nuclear morphogenesis, little is known about its influence on cellular secretion, which can regulate neighboring cells and is critical for regenerative engineering. Previous studies have shown that nuclear mechanotransduction, activated by substrate stiffening or cellular compression, can impact cell secretions.^50,51^ Here, we found that cells with deformed nuclei exhibited higher expression levels of ECM components and binding proteins that support collagen-enriched ECM organization. Additionally, soluble proteins secreted by these deformed cells were able to diffuse and modulate ECM secretion and organization in neighboring cells, as demonstrated by a transwell assay. The ECM is a complex, dynamic environment with tightly regulated mechanical and biochemical properties that affect essential cell functions, including adhesion, proliferation, and differentiation.^52^ ECM fiber alignment increases local matrix stiffness, which promotes higher force generation and increases cell stiffness, creating a positive feedback loop between cells and the matrix.^53^ Furthermore, the organized ECM enhances calcium recruitment and accelerates mineralization, contributing to effective bone regeneration.

Implantation of the flat and micropillar mPOC/HA scaffolds seeded with MSCs resulted in larger new bone volume formation *in vivo* compared to previous studies using mPOC alone, a finding likely due to the osteoconductive properties of HA. ST analysis revealed a significant upregulation of genes encoding cartilage oligomeric matrix protein (COMP) and fibromodulin (FMOD) in the micropillar group, consistent with the secretome analysis. COMP binds to matrix proteins like collagen, enhancing ECM organization and assembly.^54^ As an ECM protein, COMP also promotes osteogenesis by binding to bone morphogenetic protein 2 (BMP-2), increasing its local concentration and boosting its biological activity.^55^ FMOD, with a strong affinity for the HA matrix, helps attenuate osteoclast precursor maturation, thereby influencing osteoblast–osteoclast crosstalk.^56^ These results suggest that nuclear deformation induced by micropillars may promote osteogenesis in neighboring cells via matricrine effects.

Despite the enhanced bone regeneration observed, mPOC/HA implants did not achieve complete healing of the cranial defect, likely due to the limited interaction surface of the film scaffold. The influence of the implants, whether through direct chromatin reprogramming guidance or secretome activity, was restricted to cells at the tissue-scaffold interface. Future efforts should focus on the design and fabrication of 3D micropillar implants using additive manufacturing and composite materials to create a more comprehensive 3D cellular microenvironment that promotes bone regeneration. Additionally, the application of micropillars as a platform for delivering bioactive factors could be explored as a strategy to achieve complete cranial bone healing.

In summary, we investigated the effects of nuclear deformation on the cellular secretome using micropillar implants fabricated from an mPOC/HA composite. The mPOC/HA micropillars demonstrated similar properties to a flat substrate in terms of roughness and degradation but had a substantial impact on cellular and nuclear morphology, cell adhesion, cytoskeletal development, and osteogenic differentiation in hMSCs. Nuclear-deformed cells showed increased secretion of proteins and RNA transcriptions that regulate ECM components and organization, promoting osteogenesis in neighboring cells both *in vitro* and *in vivo*. These findings suggest that incorporating microtopography into implants holds significant promise for bone regeneration. This study offers valuable insights for the future design and fabrication of bioactive implants in regenerative engineering.

## Materials and Methods

### Synthesis and characterization of mPOC pre-polymer.

The mPOC pre-polymer were synthesized according to a previous report.^32^ Briefly, the POC pre-polymer was firstly synthesized by reaction of equal molar of citric acid (Sigma-Aldrich, 251275) and 1,8-octandiol (Sigma-Aldrich, O3303) at 140 °C oil bath for 60 min. The product was then purified by precipitation in DI water. After lyophilization, 66g POC pre-polymer was dissolved in 540 ml tetrahydrofuran (THF) and reacted with 0.036 mol imidazole (Sigma-Aldrich, I2399) and 0.4 mol glycidyl methacrylate (Sigma-Aldrich, 151238) at 60 °C for 6 h. The final product was then purified by precipitation in DI water and lyophilized for storage at −20 °C. Successful synthesis of mPOC pre-polymer was characterized using proton nuclear magnetic resonance (1H-NMR, Bruker A600).

### Fabrication and characterization of mPOC/HA micropillar scaffolds

SU-8 micropillar structures (5×5×8 um^3^) were fabricated according to our previous study.^13^ PDMS molds were then fabricated to replicate the invert structures. HA nanoparticles (Sigma-Aldrich, 677418) were mixed with mPOC pre-polymer at weight ratio of 6:4. The 60% HA was selected to mimic composition of native bone.^57^ Photo-initiator (5 mg/ml camphorquinone and ethyl 4-dimethylaminobenzoate) was added to the mPOC/HA slurry. The mixture was then added onto PDMS mold and pressed onto cover glass to prepare free-standing scaffold under exposure with laser (1W, 470 nm). Post-curing of the scaffold was performed in 80 °C oven over night. The size of HA nanoparticles was characterized using Dynamic Light Scattering (DLS). The topography of micropillars was observed using scanning electron microscope (SEM, FEI Quanta 650 ESEM) and characterized using 3D optical microscope (Bruker). Surface roughness of flat and micropillar scaffolds was characterized using atomic force microscope (AFM, Bruker ICON system). The water contact angle was tested using VCA Optima XE system. The compressive modulus of the scaffolds was characterized using a Tribioindenter (Bruker). Based on a previous report,^58^ the lateral modulus of micropillars was calculated according to the following equations:

(1) $$k_{L}=\frac{3EI}{L^{3}}$$

The $k_{L}$ is the lateral stiffness, ‘E’ is the measured modulus, ‘I’ is the moment area of inertia, and ‘L’ is the micropillar height. For square micropillars, ‘I’ can be described as:

(2) $$I=\frac{a^{4}}{12}$$

Where ‘a’ is the side length of the micropillars. Thus, the lateral modulus of the micropillars ‘$E_{L}$’ equals to:

(3) $$E_{L}=\frac{K_{L}L}{A}$$

Where ‘A’ is the cross-section area of micropillars.

### Degradation and calcium release

To test the degradation of the mPOC/HA scaffold, the dry weight of mPOC/HA scaffolds at day 0 was recorded as the initial weight. Then the scaffolds were merged in 1 ml DPBS solution in 75 °C oven. At each designed time point (1, 2, 3, 5, 7, 10 and 14 d), the scaffolds were rinsed with DI water followed by drying at 60 °C. The weight was recorded to calculate the weight loss percentage. The calcium release test was also performed with 75 °C DPBS (no calcium, no magnesium). At the designed time points, the elution solution was collected and replaced with fresh DPBS (1 ml). The released calcium was detected with inductively coupled plasma mass spectrometry (ICP-MS, ThermoFisher Element 2). Accumulated calcium release was calculated.

### Cell culture

Human mesenchymal stromal cells (hMSCs, PCS-500-012) were purchased from the American Type Culture Collection (ATCC) and cultured with the growth medium acquired from ATCC. hMSCs with the passage 4–6 were seeded onto the flat and micropillar mPOC/HA substrates. To test cell attachment, hMSCs were seeded at 5000 cells/cm^2^ and cultured for 3 h followed by PBS rinsing to remove unattached cells. The attached cells were then trypsinized and collected for cell counting. For other experiments, the cells were cultured in growth medium for 24 h to allow cell attachment and spreading followed by incubation with osteogenic induction medium. After 3 d culture, live/dead staining (Thermofisher, L3224), MTT assay (Thermofisher, V13154), and Picogreen assay (Thermofisher, P7589) were performed according to the manufactures’ protocol.

### Nuclear morphology analysis

After one day of culture, the cells were fixed with 4% paraformaldehyde, and cell nuclei were stained using SYTOX^™^ Green (ThermoFisher, S7020) according to the manufacture’s instruction. The nuclear shape index (NSI) was analyzed to evaluate 2D nuclear deformation.^27^ The stained cells were then imaged using a confocal microscope (Leica SP8) to acquire their 3D morphology. Cell nuclei were reconstructed using the Fiji ImageJ software (https://imagej.net/Fiji). Cell nuclear volume, surface area, project area, height, and the ratio of surface area to volume were measured using 3D objects counter plugin. More than 30 nuclei from 3 biological replicates were imaged and analyzed to calculate the statistics.

### Scanning electron microscope

To visualize cell adhesion on mPOC/HA scaffolds, cells were fixed with 3% glutaraldehyde (Electron Microscopy Sciences) and rinsed with DI water. Subsequently, the cells underwent dehydration using a series of ethanol concentrations (30%, 50%, 70%, 90%, and 100%) for 5 min each, followed by drying using a critical point dryer (Tousimis Samdri) as per the manual. The dehydrated cells were coated with a 5 nm osmium layer and imaged using a scanning electron microscope (SEM, FEI Quanta 650). Captured images were further enhanced for visualization of cellular architecture using Photoshop. Additionally, cells on transwell were imaged using SEM and EDS analysis was performed to evaluate the calcium and phosphate deposition.

### Osteogenic differentiation

hMSCs were seeded onto both flat and micropillar mPOC/HA substrates. One-day post-seeding, osteogenic induction medium (Lonza) was applied to prompt the osteogenic differentiation of hMSCs. After 7 days of induction, cells were washed with PBS buffer and fixed with 4% paraformaldehyde for 10 minutes. Subsequently, the samples were immersed in a solution of 56 mM 2-amino-2-methyl-1,3-propanediol (AMP, pH~9.9), containing 0.1% naphthol AS-MX phosphate and 0.1% fast blue RR salt to stain alkaline phosphatase (ALP). Bright-field images were acquired using a Nikon Eclipse TE2000-U inverted microscope. ALP activity was assessed using the ALP assay kit (K422-500, Biovision) following the provided manual. Briefly, cells cultured in induction medium for 7 days were homogenized using ALP assay buffer. Subsequently, the non-fluorescent substrate 4-Methylumelliferyl phosphate disodium salt (MUP) was mixed with the homogenized samples to generate a fluorescent signal through its cleavage by ALP. Fluorescence intensity was measured using a Cytation 5 imaging reader (BioTek) at (Ex/Em = 360/440 nm). Enzymatic activity was calculated based on the standard curve and normalized to total DNA content, determined by the Quant-iT PicoGreen dsDNA assay (Invitrogen). The expression levels of OCN and RUNX2 were quantified through Western Blot analysis. In brief, cell lysis was performed using radioimmunoprecipitation assay (RIPA) buffer. The relative protein quantities were measured using a Cytation 5 imaging reader. Equal amounts of proteins extracted from flat and micropillar samples were loaded onto a NuPAGE 4–12% Bis-Tris Gel (Invitrogen) and subsequently transferred to nitrocellulose membranes (Bio-rad). Afterward, membranes were blocked with 5% milk and incubated with primary antibodies (including GAPDH from Abcam, OCN from Cell Signaling, RUNX2 from Santa Cruz) overnight at 4 °C with gentle shaking. Following this, secondary antibodies, diluted at a ratio of 1:5000, were applied and incubated with the membranes at room temperature for 1 hour. Protein bands were visualized using the Azure 600 gel imaging system. The acquired images underwent analysis through the ‘Gel Analyzer’ tool in ImageJ. The intensity of all target protein bands was initially compared to the corresponding GAPDH, and then normalized against a flat surface, which was set as 1. Statistical calculations were based on three biological replicates.

### Secretome sample preparation:

Analysis of secreted proteins is complicated by high concentrations of serum proteins. Our approach reduced initial sample volume to a 20 μl concentrate using a molecular weight cut off filter (50 kDa, Amicon Ultra-15 centrifugal, Ultracel, Merck). The concentrate above 50KDa was depleted of the most abundant proteins using a High Select HAS / Immunoglobulin Depletion Midi spin column (A36367, Thermo Fisher Scientific), resulting in a filtrate solution (below 50KDa) and a depleted solution per sample. An acetone / TCA (Trichloroacetic acid) protein precipitation was performed on each solution to create protein pellets and an in-solution trypsin digestion was performed on each pellet.100 μl of re-suspension buffer (8 M urea in 400 mM ammonium bicarbonate) was added to the pellet and incubated with mixing for 15 minutes. Disulfide bonds were reduced by addition of 100 mM dithiothreitol and incubated for 45 minutes at 55 °C. Sulfhydryl groups were alkylated by addition of 300 mM iodoacetamide and incubated for 45 minutes at 25 °C shielded from light. Samples were diluted 4-fold with ammonium bicarbonate to reduce the urea concentration below 2 M. Protein digestion was performed by addition of trypsin (MS-grade, Promega) at a 1:50 ratio (enzyme:substrate) and incubated overnight at 37 °C. Digestion was halted with the addition of 10 % formic acid (FA) to a final concentration of 0.5%. Peptides were desalted with C18 spin columns (The Nest Group), dried by vacuum centrifugation, and stored at −20 °C. Peptides were resuspended in 5% ACN (Acetonitrile) / 0.1% FA for LC-MS analysis. Peptide concentration was quantified using micro BCA (Bicinchoninic acid) protein assay kit (Thermo Scientific, Ref: 23235).

### Proteome sample preparation:

Cells were lysed using cell lysis buffer (0.5% SDS, 50mM Ambic (Ammonium Bicarbonate), 50mM NaCl (Sodium Chloride), Halt Protease inhibitor). An acetone / TCA protein precipitation was performed on each lysed samples solution to create protein pellets and an in-solution trypsin digestion was performed on each pellet. 100 μl of re-suspension buffer (8 M urea in 400 mM ammonium bicarbonate) was added to the pellet and incubated with mixing for 15 minutes. Disulfide bonds were reduced by addition of 100 mM dithiothreitol and incubated for 45 minutes at 55 °C. Sulfhydryl groups were alkylated by addition of 300 mM iodoacetamide and incubated for 45 minutes at 25 °C shielded from light. Samples were diluted 4-fold with ammonium bicarbonate to reduce the urea concentration below 2 M. Protein digestion was performed by addition of trypsin (MS-grade, Promega) at a 1:50 ratio (enzyme:substrate) and incubated overnight at 37 °C. Digestion was halted with the addition of 10 % formic acid to a final concentration of 0.5%. Peptides were desalted with C18 spin columns (The Nest Group), dried by vacuum centrifugation, and resuspended in 5% ACN/0.1% FA for LC-MS analysis. Peptide concentration was quantified using micro BCA Protein Assay Kit (Thermo Scientific, Ref: 23235).

### Liquid Chromatography High Resolution Tandem Mass Spectrometry (LC-HRMS/MS) Analysis:

Peptides were analyzed using a Vanquish Neo nano-LC coupled to a Exploris 480 hybrid quadrupole-orbitrap mass spectrometer (Thermo Fisher Scientific, USA). The samples were loaded onto the trap column of 75μm internal diameter (ID) × 2cm length (Acclaim PepMap^™^ 100, P/N 164535) and analytical separation was performed using a UHPLC C18 column (15cm length × 75μm internal diameter, 1.7μm particle size, Ion Opticks, AUR3-15075C18). For each run, 1 μg of peptide sample was injected. Electrospray ionization was performed using a Nanospray Flex Ion Source (Thermo Fisher, ES071) at a positive static spray voltage of 2.3 kV. Peptides were eluted from the analytical column at a flow rate of 200 nL / min using an increasing organic gradient to separate peptides based on their hydrophobicity. Buffer A was 0.1 % formic acid in Optima LC-MS grade water, and buffer B was 80 % acetonitrile, 19.9 % Optima LC-MS grade water, and 0.1 % formic acid: The method duration was 120 minutes. The mass spectrometer was controlled using Xcalibur and operated in a positive polarity. The full scan (MS1) settings used were: mass range 350–2000 m/z, RF lens 60 %, orbitrap resolution 120,000, normalized AGC target 300 %, maximum injection time of 25 milliseconds, and a 5E^3^ intensity threshold. Data-dependent acquisition (DDA) by TopN was performed through higher-energy collisional dissociation (HCD) of isolated precursor ions with charges of 2+ to 5+ inclusive. The MS2 settings were: dynamic exclusion mode duration 30 seconds, mass tolerance 5 ppm (both low and high), 2 second cycle time, isolation window 1.5 m/z, 30 % normalized collision energy, orbitrap resolution 15,000, normalized AGC target 100 %, and maximum injection time of 50 milliseconds.

### Data analysis:

Mass spectrometry files (.raw) were converted to Mascot generic format (.mgf) using the Scripps RawConverter program and then analyzed using the Mascot search engine (Matrix Science, version 2.5.1). MS/MS spectra were searched against the SwissProt database of the organism of interest. Search parameters included a fixed modification of cysteine carbamidomethylation, and variable modifications of methionine oxidation, deamidated asparagine and aspartic acid, and acetylated protein N-termini. Two missed tryptic cleavages were permitted. A 1 % false discovery rate (FDR) cutoff was applied at the peptide level. Only proteins with at least two peptides were considered for further study.

### Label-Free Quantification:

The samples were acquired on mass spec and the data were searched against a specific database using the MaxQuant application.^59^ Label-Free Quantification (LFQ) was obtained by LFQ MS1 intensity. The results were filtered with a minimum of 2 unique peptides. Technical replicates were averaged and intensities were Log2 transformed to achieve a normal distribution of the data. Missing values were filtered to keep only proteins quantified in at least 2 samples per group. For statistics, Student t-Test was applied using p < 0.05 and FC > 2 to determine which proteins were significantly up- and down-regulated and visualize it by volcano plot. Downstream analyses and visualizations were done using RStudio software (R version 4.3.2, RStudio version 2024.09.0). Principal component analysis (PCA) was done using ‘prcomp’ R function to visualize la ability of the differential protein expression to distinguish between biological conditions. Heatmap plot was built using ‘ComplexHeatmap’ R package. GO and Pathways enrichment analysis was done using ‘clusterProfiler’ R package^60^ and annotations with adjusted p-values (FDR, Benjamini-Hochberg) < 0.05 were considered significant. Additional packages used include ‘org.Hs.eg.db’ for human gene annotations and ‘enrichplot’ for visualization. This analysis considered the entire set of human protein-coding genes as the reference background.

### Transwell assay:

The flat and micropillar mPOC/HA surfaces were fabricated in a 24 well plate. The hMSCs were seeded onto the surfaces with 40,000 cells per well. Then a transwell was put in each well and additional hMSCs were seeded inside the transwell (Costar, 0.4 μm polyester membrane) at density of 5,000 cells/cm^2^. After cell attachment, osteogenic medium was used to induce osteogenic differentiation of the cells. At 7 days post-induction, the cells on transwell were fixed followed by ALP staining and quantification to investigate the paracrine effect of deformed and undeformed cells on osteogenesis. At 3 weeks post-induction, additional transwells were collected for Alizarin Red S (ARS) staining and quantification to show the calcium deposition influenced by the paracrine effect. At 4 weeks post-induction, the collagen, which is one of the major components in ECM and significantly affected according to the secretome analysis, were stained using anti-collagen antibody (Abcam, ab36064) to investigate the influence of nuclear deformation on ECM organization.

### In vivo implantation:

The animal study was approved by the University of Chicago Animal Care and Use Committee following NIH guidance (ACUP#71745). Eight-week-old female athymic nude mice obtained from Harlan Laboratories were used for the study. The animals were housed in a separately air-conditioned cabinet at temperature of 24–26 °C with 12:12 light:dark cycle. The surgeries were performed according to the previous report61. Briefly, animals were treated with 2% isoflurane delivered by 100% O2 and maintained with 1–1.5% isoflurane for anaesthesia. Two critical-sized defects (4 mm diameter) were created on the left and right side of skull of each animal followed by implantation of hMSCs seeded flat and micropillar scaffolds, respectively. After implantation of scaffolds, a larger mPOC film (1 × 1.5 cm2) was attached to the skull with thrombin/fibrinogen to prevent displacement of implants. Skin tissue was closed with 5–0 nylon interrupted sutures and removed after 2 weeks. The animals were monitored after anaesthesia hourly until recovery. Buprenorphine 50 μg kg^−1^ and meloxicam 1 mg kg^−1^ were used for pain relief.

### Micro-CT:

Micro-CT images of cranial were performed on the XCUBE (Molecubes NV) by the Integrated Small Animal Imaging Research Resource (iSAIRR) at The University of Chicago. Spiral high-resolution computed tomography acquisitions were performed with an X-ray source of 50 kVp and 440 μA. Volumetric computed tomography images were reconstructed by applying the iterative image space reconstruction algorithm (ISRA) in a 400 × 400 × 370 format with voxel dimensions of 100 × 100 × 100 μm^3^. An Amira software (Thermo Scientific) was used for 3D reconstruction of the skull tissue and to analyse the bone formation in the defect area. Scale bars were used to standardize the images. Defect recovery is defined as (Vi − Vd)/Vi × 100%, where Vi and Vd represent defect volume at initial and designed timepoints, respectively.

### Histology analysis:

Skull samples were fixed and decalcified in Cal-EX II (Fisher Scientific) for 24 hours, rinsed with PBS, and embedded in paraffin. Tissue sections containing defect sites were cut to 5 μm thickness and stained with H&E and trichrome to assess tissue regeneration. Regenerated tissue thickness was measured using ImageJ, and osteogenesis was evaluated via IHC staining for key osteogenic markers, including OCN and OPN. Mouse skin tissue served as a negative control for all IHC staining.

### Spatial transcriptomics:

To confirm the RNA quality of each FFPE tissue block, 1–2 curls (10um thickness each) were used for RNA extraction using Qiagen RNeasy FFPE kit (Qiagen 73504) according to manufactures’ protocol. Extracted RNA was examined by Agilent Bioanalyzer RNA pico chip to confirm the DV200 >30%. Simultaneously, the tissue morphology was examined on HE stained slide to identify region of interest.

For each FFPE sample, 1 section (5um thickness) was placed on visium slides. Each slide was incubated at 42°C for 3 hours followed by overnight room temperature incubation. Then, the slide was stored at desiccated slide holder until proceeding to deparaffinization.

The deparaffinization, HE staining and imaging and decrosslinking of tissue slides were performed according to 10x Genomics protocol (CG000409 and CG000407) specific for Visium spatial gene expression for FFPE kit. Then, the slides were proceeded to human probe (v2) hybridization and ligation using 10x Genomics Visium spatial gene expression, 6.5mm kit (10x Genomics, PN-1000188). The probes were released from tissue slide and captured on visium slide followed by probe extension. Sequencing libraries were prepared according to manufacturer’s protocol. Multiplexed libraries were pooled and sequenced on Novaseq X Plus 10Bflowcell 100 cycles kit with following parameter: 28nt for Read 1 and 90nt for Read 2.

We visually identified the implant region in each sample. To exclude low quality capture locations, we removed the capture locations with fewer than 500 unique molecular identifiers, fewer than 500 genes, or ⩾ 25% mitochondrial reads.^61^ We also filtered out the genes that are expressed in fewer than five capture locations.^61^ After quality control, flat group had 101 capture locations and 12,701 genes, whereas micropillar group had 73 capture locations and 13,371 genes.

### Differential gene expression analysis:

To identify the genes differentially expressed in flat and micropillar groups, we performed Wilcoxon rank-sum tests on the merged dataset (174 capture locations) using the FindAllMarkers function in Seurat V3.^62^ Our testing was limited to the genes present in both implants, detected in a minimum 1% of cells in either implant, as well as showing at least 0.1 log-fold difference between the two implants.

### Cell type deconvolution:

To perform cell typing on our data, we first identified three publicly available bone single-cell RNA sequencing (scRNA-seq) references with annotated cell types.^43–45^ The scRNA-seq references were processed, quality controlled, and merged using Seurat V3. Since our samples are nude mice, we excluded all the immune cells from the merged reference. The final merged scRNA-seq dataset contained a total of 12,717 cells and represented all major cell types present in bone tissues.

In 10x Visium data, each capture location contains a mixture of cells.^63^ Therefore, we performed cell type deconvolution to predict the cell type proportions in each capture location using BayesPrism, a Bayesian deconvolution method shown to work on spatial transcriptomics data.^64,65^ We excluded chromosomes X and Y, ribosomal, and mitochondrial genes from the analysis to reduce batch effects. We also removed the outlier genes with expression greater than 1% of the total reads in over 10% of capture locations. To improve cell typing accuracy, we only used the cell type signature genes for deconvolution analysis. The cell type markers were identified based on the differential expression analysis results on the merged scRNA-seq reference. The predicted cell type proportions with above 0.5 coefficient of variation were clipped to zero to reduce noise.

### Cell-type-based analyses:

We performed Wilcoxon rank-sum tests using the deconvoluted cell type proportions to test if certain cell types are more prevalent in one implant than the other. We further examined the association between cell type proportions and gene expression levels in the two implants through Kendall’s correlation analyses. All the p-values were adjusted for multiple testing through the false discovery rate approach. The proportions of three cell types (chondrocyte, OLC, and osteocyte) had over 50 significantly positively correlated genes. For each of these cell types, we performed pathway enrichment analysis of the significantly positively correlated genes using Metascape.^66^

### Statistical analysis:

The results are shown as mean ± standard deviation using violin super plots or bar graphs. Statistical analysis was performed using Kyplot software (version 2.0 beta 15). Statistical significance was determined by Student’s t-test (flat versus micropillar, two-sided). All experiments presented in the manuscript were repeated at least as two independent experiments with replicates to confirm the results are reproducible.

## References

[1] Rippe K. Dynamic organization of the cell nucleus. Curr. Opin. in Genet. Dev. 17, 373–380 (2007). 17913491 10.1016/j.gde.2007.08.007

[2] Kalukula Y., Stephens A. D., Lammerding J. & Gabriele S. Mechanics and functional consequences of nuclear deformations. Nat. Rev. Mol. Cell Biol. 23, 583–602 (2022). 35513718 10.1038/s41580-022-00480-z PMC9902167

[3] Ramdas N. M. & Shivashankar G. V. Cytoskeletal Control of Nuclear Morphology and Chromatin Organization. J. Mol. Biol. 427, 695–706 (2015). 25281900 10.1016/j.jmb.2014.09.008

[4] Heckenbach I. Nuclear morphology is a deep learning biomarker of cellular senescence. Nat. Aging 2, 742–755 (2022). 37118134 10.1038/s43587-022-00263-3 PMC10154217

[5] Seelbinder B. Nuclear deformation guides chromatin reorganization in cardiac development and disease. Nat. Biomed. Eng. 5, 1500–1516 (2021). 34857921 10.1038/s41551-021-00823-9 PMC9300284

[6] Uhler C. & Shivashankar G. V. Nuclear Mechanopathology and Cancer Diagnosis. Trends in cancer 4, 320–331 (2018). 29606315 10.1016/j.trecan.2018.02.009

[7] Lele T. P., Levy D. L. & Mishra K. Editorial: Nuclear morphology in development and disease. Front. Cell Dev. Biol. 11 (2023). 10.3389/fcell.2023.1267645 PMC10443097 37614225

[8] Dahl K. N., Ribeiro A. J. & Lammerding J. Nuclear shape, mechanics, and mechanotransduction. Circ. Res. 102, 1307–1318 (2008). 18535268 10.1161/CIRCRESAHA.108.173989 PMC2717705

[9] Wang X. Mechanical stability of the cell nucleus – roles played by the cytoskeleton in nuclear deformation and strain recovery. J. Cell Sci. 131 (2018). 10.1242/jcs.209627 29777038

[10] Elosegui-Artola A. Force Triggers YAP Nuclear Entry by Regulating Transport across Nuclear Pores. Cell 171, 1397–1410.e1314 (2017). 29107331 10.1016/j.cell.2017.10.008

[11] Lomakin A. J. The nucleus acts as a ruler tailoring cell responses to spatial constraints. Science 370, eaba2894 (2020). 33060332 10.1126/science.aba2894 PMC8059074

[12] Venturini V. The nucleus measures shape changes for cellular proprioception to control dynamic cell behavior. Science 370, eaba2644 (2020). 33060331 10.1126/science.aba2644

[13] Wang X. Chromatin reprogramming and bone regeneration in vitro and in vivo via the microtopography-induced constriction of cell nuclei. Nat. Biomed. Eng. 7, 1514–1529 (2023). 37308586 10.1038/s41551-023-01053-x PMC10804399

[14] Liu H. In Situ Mechanical Characterization of the Cell Nucleus by Atomic Force Microscopy. ACS Nano 8, 3821–3828 (2014). 24673613 10.1021/nn500553z

[15] Kechagia Z. The laminin–keratin link shields the nucleus from mechanical deformation and signalling. Nat. Mater. 22, 1409–1420 (2023). 37709930 10.1038/s41563-023-01657-3 PMC10627833

[16] Wang X. Intracellular manipulation and measurement with multipole magnetic tweezers. Sci. Robot. 4, eaav6180 (2019). 33137746 10.1126/scirobotics.aav6180

[17] Hwang J. Y. Cell Deformation by Single-beam Acoustic Trapping: A Promising Tool for Measurements of Cell Mechanics. Sci. Rep. 6, 27238 (2016). 27273365 10.1038/srep27238 PMC4897707

[18] Stöberl S. Nuclear deformation and dynamics of migrating cells in 3D confinement reveal adaptation of pulling and pushing forces. Sci. Adv. 10, eadm9195 (2024). 39167661 10.1126/sciadv.adm9195 PMC11338266

[19] Song Y. Transient nuclear deformation primes epigenetic state and promotes cell reprogramming. Nat. Mater. 21, 1191–1199 (2022). 35927431 10.1038/s41563-022-01312-3 PMC9529815

[20] Shah P. Nuclear Deformation Causes DNA Damage by Increasing Replication Stress. Curr. Biol. 31, 753–765.e756 (2021). 33326770 10.1016/j.cub.2020.11.037 PMC7904640

[21] Hanson L. Vertical nanopillars for in situ probing of nuclear mechanics in adherent cells. Nat. Nanotechnol. 10, 554–562 (2015). 25984833 10.1038/nnano.2015.88 PMC5108456

[22] Davidson P. M., Özçelik H., Hasirci V., Reiter G. & Anselme K. Microstructured Surfaces Cause Severe but Non-Detrimental Deformation of the Cell Nucleus. Adv. Mater. 21, 3586–3590 (2009).

[23] Tusamda Wakhloo N. Actomyosin, vimentin and LINC complex pull on osteosarcoma nuclei to deform on micropillar topography. Biomaterials 234, 119746 (2020). 31945617 10.1016/j.biomaterials.2019.119746

[24] Cao X. A Chemomechanical Model for Nuclear Morphology and Stresses during Cell Transendothelial Migration. Biophys. J. 111, 1541–1552 (2016). 27705776 10.1016/j.bpj.2016.08.011 PMC5052451

[25] Liu R., Yao X., Liu X. & Ding J. Proliferation of Cells with Severe Nuclear Deformation on a Micropillar Array. Langmuir : the ACS journal of surfaces and colloids 35, 284–299 (2019). 30513205 10.1021/acs.langmuir.8b03452

[26] Carthew J. Precision Surface Microtopography Regulates Cell Fate via Changes to Actomyosin Contractility and Nuclear Architecture. Adv. Sci. 8, 2003186 (2021). 10.1002/advs.202003186 PMC7967085 33747730

[27] Liu X. Subcellular cell geometry on micropillars regulates stem cell differentiation. Biomaterials 111, 27–39 (2016). 27716524 10.1016/j.biomaterials.2016.09.023

[28] Long Y., Sun Y., Jin L., Qin Y. & Zeng Y. Micropillars in Biomechanics: Role in Guiding Mesenchymal Stem Cells Differentiation and Bone Regeneration. Adv. Mater. Interfaces 11, 2300703 (2024).

[29] Xu H. Citric Acid: A Nexus Between Cellular Mechanisms and Biomaterial Innovations. Adv. Mater. 36, 2402871 (2024). 10.1002/adma.202402871 PMC11309907 38801111

[30] Epstein S. E., Luger D. & Lipinski M. J. Paracrine-Mediated Systemic Anti-Inflammatory Activity of Intravenously Administered Mesenchymal Stem Cells. Circ. Res. 121, 1044–1046 (2017). 29025759 10.1161/CIRCRESAHA.117.311925

[31] Burdon T. J., Paul A., Noiseux N., Prakash S. & Shum-Tim D. Bone Marrow Stem Cell Derived Paracrine Factors for Regenerative Medicine: Current Perspectives and Therapeutic Potential. Bone Marrow Res. 2011, 207326 (2011). 22046556 10.1155/2011/207326 PMC3195349

[32] Wang Y., Kibbe M. R. & Ameer G. A. Photo-crosslinked biodegradable elastomers for controlled nitric oxide delivery. Biomater. Sci. 1, 625–632 (2013). 24707352 10.1039/C3BM00169E PMC3972038

[33] Fernando S., McEnery M. & Guelcher S. A. in Advances in Polyurethane Biomaterials (eds Cooper Stuart L.& Guan Jianjun) 481–501 (Woodhead Publishing, 2016).

[34] Ghibaudo M. Traction forces and rigidity sensing regulate cell functions. Soft Matter 4, 1836–1843 (2008).

[35] Badique F. Directing nuclear deformation on micropillared surfaces by substrate geometry and cytoskeleton organization. Biomaterials 34, 2991–3001 (2013). 23357373 10.1016/j.biomaterials.2013.01.018

[36] Ryu H. Materials and Design Approaches for a Fully Bioresorbable, Electrically Conductive and Mechanically Compliant Cardiac Patch Technology. Adv. Sci. 10, 2303429 (2023). 10.1002/advs.202303429 PMC10520666 37518771

[37] Khalili A. A. & Ahmad M. R. A Review of Cell Adhesion Studies for Biomedical and Biological Applications. Int. J. Mol. Sci 16, 18149–18184 (2015). 26251901 10.3390/ijms160818149 PMC4581240

[38] Tomczak A. Interpretation of biological experiments changes with evolution of the Gene Ontology and its annotations. Sci. Rep. 8, 5115 (2018). 29572502 10.1038/s41598-018-23395-2 PMC5865181

[39] Fabregat A. Reactome pathway analysis: a high-performance in-memory approach. BMC Bioinformatics 18, 142 (2017). 28249561 10.1186/s12859-017-1559-2 PMC5333408

[40] Lu P., Takai K., Weaver V. M. & Werb Z. Extracellular matrix degradation and remodeling in development and disease. Cold Spring Harb Perspect Biol. 3 (2011). 10.1101/cshperspect.a005058 PMC3225943 21917992

[41] Zeng Z., Li Y., Li Y. & Luo Y. Statistical and machine learning methods for spatially resolved transcriptomics data analysis. Genome Biol. 23, 83 (2022). 35337374 10.1186/s13059-022-02653-7 PMC8951701

[42] Yu M. Cranial Suture Regeneration Mitigates Skull and Neurocognitive Defects in Craniosynostosis. Cell 184, 243–256.e218 (2021). 33417861 10.1016/j.cell.2020.11.037 PMC7891303

[43] Dillard L. J. Single-Cell Transcriptomics of Bone Marrow Stromal Cells in Diversity Outbred Mice: A Model for Population-Level scRNA-Seq Studies. J. Bone Miner. Res. 38, 1350–1363 (2023). 37436066 10.1002/jbmr.4882 PMC10528806

[44] Han X. Mapping the Mouse Cell Atlas by Microwell-Seq. Cell 172, 1091–1107.e1017 (2018). 29474909 10.1016/j.cell.2018.02.001

[45] Baryawno N. A Cellular Taxonomy of the Bone Marrow Stroma in Homeostasis and Leukemia. Cell 177, 1915–1932.e1916 (2019). 31130381 10.1016/j.cell.2019.04.040 PMC6570562

[46] Zhong L. Single cell transcriptomics identifies a unique adipose lineage cell population that regulates bone marrow environment. eLife 9, e54695 (2020). 32286228 10.7554/eLife.54695 PMC7220380

[47] Ma C. Citrate-based materials fuel human stem cells by metabonegenic regulation. Proc. Natl. Acad. Sci. USA 115, E11741–E11750 (2018). 30478052 10.1073/pnas.1813000115 PMC6294936

[48] Woodard J. R. The mechanical properties and osteoconductivity of hydroxyapatite bone scaffolds with multi-scale porosity. Biomaterials 28, 45–54 (2007). 16963118 10.1016/j.biomaterials.2006.08.021

[49] Wang H., Huddleston S., Yang J. & Ameer G. A. Enabling Proregenerative Medical Devices via Citrate-Based Biomaterials: Transitioning from Inert to Regenerative Biomaterials. Adv. Mater. 36, 2306326 (2024). 10.1002/adma.202306326 38043945

[50] Vilar A. Substrate mechanical properties bias MSC paracrine activity and therapeutic potential. Acta Biomater. 168, 144–158 (2023). 37422008 10.1016/j.actbio.2023.06.041

[51] Li Y. 3D micropattern force triggers YAP nuclear entry by transport across nuclear pores and modulates stem cells paracrine. Natl. Sci. Rev. 10 (2023). 10.1093/nsr/nwad165 PMC10347367 37457331

[52] Karamanos N. K. A guide to the composition and functions of the extracellular matrix. The FEBS J. 288, 6850–6912 (2021). 33605520 10.1111/febs.15776

[53] Saraswathibhatla A., Indana D. & Chaudhuri O. Cell–extracellular matrix mechanotransduction in 3D. Nat. Rev. Mol. Cell Biol. 24, 495–516 (2023). 36849594 10.1038/s41580-023-00583-1 PMC10656994

[54] Cui J. & Zhang J. Cartilage Oligomeric Matrix Protein, Diseases, and Therapeutic Opportunities. Int. J. Mol. Sci. 23, 9253 (2022). 36012514 10.3390/ijms23169253 PMC9408827

[55] Ishida K. Cartilage oligomeric matrix protein enhances osteogenesis by directly binding and activating bone morphogenetic protein-2. Bone 55, 23–35 (2013). 23528838 10.1016/j.bone.2013.03.007

[56] Zheng Z., Granado H. S. & Li C. Fibromodulin, a Multifunctional Matricellular Modulator. J. Dent. Res. 102, 125–134 (2023). 36515321 10.1177/00220345221138525 PMC9986681

[57] Feng X. Chemical and Biochemical Basis of Cell-Bone Matrix Interaction in Health and Disease. Curr. Chem. Biol. 3, 189–196 (2009). 20161446 10.2174/187231309788166398 PMC2790195

[58] Alapan Y., Younesi M., Akkus O. & Gurkan U. A. Anisotropically Stiff 3D Micropillar Niche Induces Extraordinary Cell Alignment and Elongation. Adv. Healthc. Mater. 5, 1884–1892 (2016). 27191679 10.1002/adhm.201600096 PMC4982772

[59] Cox J. & Mann M. MaxQuant enables high peptide identification rates, individualized p.p.b.-range mass accuracies and proteome-wide protein quantification. Nat. Biotech. 26, 1367–1372 (2008). 10.1038/nbt.1511 19029910

[60] Yu G., Wang L. G., Han Y. & He Q. Y. clusterProfiler: an R package for comparing biological themes among gene clusters. Omics : a journal of integrative biology 16, 284–287 (2012). 22455463 10.1089/omi.2011.0118 PMC3339379

[61] Qian J. A pan-cancer blueprint of the heterogeneous tumor microenvironment revealed by single-cell profiling. Cell Res. 30, 745–762 (2020). 32561858 10.1038/s41422-020-0355-0 PMC7608385

[62] Stuart T. Comprehensive Integration of Single-Cell Data. Cell 177, 1888–1902.e1821 (2019). 31178118 10.1016/j.cell.2019.05.031 PMC6687398

[63] Li B. Benchmarking spatial and single-cell transcriptomics integration methods for transcript distribution prediction and cell type deconvolution. Nat. Methods 19, 662–670 (2022). 35577954 10.1038/s41592-022-01480-9

[64] Chu T., Wang Z., Pe’er D. & Danko C. G. Cell type and gene expression deconvolution with BayesPrism enables Bayesian integrative analysis across bulk and single-cell RNA sequencing in oncology. Nat. Cancer 3, 505–517 (2022). 35469013 10.1038/s43018-022-00356-3 PMC9046084

[65] Niec R. E. Lymphatics act as a signaling hub to regulate intestinal stem cell activity. Cell Stem Cell 29, 1067–1082.e1018 (2022). 35728595 10.1016/j.stem.2022.05.007 PMC9271639

[66] Zhou Y. Metascape provides a biologist-oriented resource for the analysis of systems-level datasets. Nat. Commun. 10, 1523 (2019). 30944313 10.1038/s41467-019-09234-6 PMC6447622

